# Exploring integrated care for children with cerebral palsy: a stakeholder analysis

**DOI:** 10.1186/s12913-025-13015-x

**Published:** 2025-07-07

**Authors:** Silje Askeland, Veslemøy Guise, Karina Aase, Maren Kristine Raknes Sogstad

**Affiliations:** 1 SHARE – Centre for Resilience in Healthcare, Faculty of Health Sciences, University of Stavanger, Stavanger, Norway; 2 Centre for Care Research, NUNU Gjøvik, Gjøvik, Norway

**Keywords:** Cerebral palsy, Children and families care needs, Stakeholders, Responsibility, Coordination, Collaboration, Integrated care

## Abstract

**Background:**

Children with cerebral palsy (CP) and their families need coordinated services. Accordingly, integrated care models have been introduced as the standard for service provision. However, situations with coordination and collaboration challenges occur leading to fragmented services that do not meet the care needs of children and families. This study aimed to identify stakeholders involved in the care and follow-up of children and their families and explore their roles, responsibilities, and relationships to inform the improvement of integrated care for children with CP.

**Methods:**

A stakeholder analysis was conducted based on interviews with children, parents, and service providers; observations in multidisciplinary coordination meetings; and a review of documents. Six families with a child aged between 8 and 12 years with a primary diagnosis of CP participated. Relevant service providers were identified through family interviews and were invited to individual or focus group interviews.

**Results:**

The results identified 42 stakeholders categorized into 14 groups offering healthcare, educational, social, and family support services. Stakeholders become involved in the families’ long-term care at different times and provide services within various time spans according to specific emerging challenges. Stakeholders’ responsibilities include diagnosing and referring patients, providing short-term treatment, and providing long-term care. Additionally, responsibilities can be overlapping and unclear, and the involved stakeholders operate under different regulations and institutional contexts, leading to gaps in patient follow-up. Relationships between the stakeholders vary from full integration to no contact.

**Conclusion:**

Long-term care for children with CP and their families is complex, involving numerous stakeholders across various sectors, governed by regulations within different institutional contexts, challenging integrated care. Stakeholders’ affiliation with different sectors and their varying roles has the potential to contribute to a holistic approach. However, without clear guidance this seems hard to achieve, which may lead to a lack of collective understanding and unmet needs for children and families. There is still a need for further research on collaborative experiences among children, families, and service providers, and their impact on integrated services.

**Supplementary Information:**

The online version contains supplementary material available at 10.1186/s12913-025-13015-x.

## Background

Cerebral palsy (CP) is one of the most common lifelong childhood disabilities, with an estimated prevalence of 2.11 per 1000 live births worldwide [1, 2]. CP is a group of developmental disorders of movement and posture commonly occurring with medical, neurological, and mental/behavioral comorbidities [3, 4]. CP is characterized by shifting periods of functional improvement followed by periods of deterioration, leading to several transitional phases between care and follow-up services [5]. The complexity of the diagnosis and clinical outcomes compel the child and family to become dependent on coordinated services for health, educational support, and social care [5, 6].

Children with CP and their families benefit from easily accessible services with a holistic and long-term focus provided by multidisciplinary teams according to a coordinated and family-centered approach [7–10]. To meet this need, integrated care models offering coordinated, continuous, and easily accessible services tailored to children and their families’ needs in a cross-sector partnership have been introduced as the standard for service provision [11]. An integrated care model emphasizes vertical integration that connects primary and secondary care; horizontal integration that connects health, educational support, and social care; and longitudinal integration that connects services across the lifespan [12, 13].

Although integrated care is essential for high-quality services, the care needs of children with CP and their families are not always met [14]. For example, children and families may meet fragmented services where it is difficult to establish a shared understanding of complex situations [15]. Such situations often result in the child and family being referred to services where providers fail to take responsibility for coordinating the overall follow-up [16]. In particular, transitions between services, especially as children with CP move into adulthood, is challenging [17, 18]. These transitions require information hand over, collaboration between service providers, the child and the family, as well as clarification of responsibilities. Children and families whose care and follow-up needs are unmet may consequently experience an escalation and deterioration of the child’s problems [3], which puts additional stress on parents [10, 19], thus challenging family relationships [19].

Coordination and continuity in service provision need to be improved [20], with calls to establish a common framework founded on a shared perspective of involved stakeholders [5]. To inform the improvement of integrated care for children with CP, this study aimed to identify stakeholders involved in the care and follow-up of children and their families and to explore their roles, responsibilities, and relationships. The following research questions were proposed:

- Who are the stakeholders involved in long-term care for children with cerebral palsy?
- How can the different stakeholders’ roles, responsibilities, and relationships in long-term care be described?

## Methods

### Study design

A stakeholder analysis approach was chosen for its ability to identify stakeholders included in long-term care for children with CP and to understand their roles, responsibilities, and relationships [21]. A stakeholder is an individual or organization interested in or affected by the issue under consideration or who could influence decision-making and implementation processes [21, 22]. In this study, stakeholders were the care units and individuals with whom the children with CP and their families interact during their long-term care.

### Context

In Norway, services for children with CP and their families are provided through various municipal, regional, and governmental units, governed by four different ministries and their associated directorates. These services encompass specialist healthcare, offering assessments and treatments in hospitals, and long-term primary healthcare provided by assigned general practitioners (GPs) responsible for medical matters, physiotherapists, and occupational therapists [23, 24]. Additionally, children and family services are responsible for welfare and counseling, providing support in challenging care situations [25, 26]. Educational services include physical, social, and teaching support in kindergarten and school settings [27–29]. Furthermore, labor and social services offer financial and social support [30]. To ensure individually tailored, holistic, and coherent services, each child has the right to an assigned coordinator and individual care plan through primary healthcare services, coordinating follow-up from the various services and sectors [23–25, 30–34]. The Norwegian welfare system is designed to provide equal access to high-quality services, irrespective of individuals’ socioeconomic status or geographical location [35, 36]. This is facilitated through the universal health and social insurance coverage, regulated by the National Insurance Act and the Patient Rights Act [31, 36, 37].

### Recruitment

Families, including parents and children, were recruited from the Child and Adolescent Habilitation unit at a university hospital in Norway. A short orientation about the study was given to eligible families upon their registration for a planned consultation. Families that met the study’s inclusion criteria of having a child aged between 6–12 years with a primary diagnosis of CP and who agreed to participate were asked to provide their contact information, allowing researcher SA to contact them. This process led to the recruitment of six families. Parents gave written consent to participate. Service units and individual service providers that the families described as important during the interviews were subsequently contacted and invited to participate in individual or focus group interviews. Consequently, twenty-nine individual service providers from six different service units were recruited. Following the interviews with families and service providers, observations were conducted in scheduled multidisciplinary coordination meetings at the children’s schools. In addition, documents describing services, regulations, challenges, and care needs for the patient group were reviewed. None of the recruited participants withdraw from the study.

### Sample

Six families with children with CP who had varying gross motor function levels (Gross Motor Function Classification System level: GMFCS level), associated comorbidities, and patient histories participated in the study.

Table 1 provides an overview of the children and families.

**Table 1:** Overview of children and families included in the study

Families	Child’s age	CP diagnosis	Comorbidity
Family 1 Mother, father, and child	8	GMFCS level 1, diagnosed at the age of 4 years	Attention Deficit/Hyperactivity Disorder (ADHD), diagnosed at the age of 7 years Learning difficulties
Family 2 Mother, father, and child	12	GMFCS level 1, diagnosed at the age of 8 years	ADHD, diagnosed at the age of 7 years Asperger, diagnosed at the age of 8 years Learning difficulties
Family 3 Mother, father, and child	8	GMFCS level 3, diagnosed at the age of 1 year	Epilepsy, diagnosed at the age of 3 years Learning difficulties
Family 4 Mother, father, and child	9	GMFCS level 4, diagnosed at the neonatal stage	Epilepsy, diagnosed at the age of 1 year Cognitive disability, diagnosed at the age of 8 years
Family 5 Mother, father, and child	9	GMFCS level 3, diagnosed at the neonatal stage	Epilepsy, diagnosed at the age of 2 years Learning difficulties
Family 6 Mother, father, and child	9	GMFCS level 1, diagnosed at the neonatal stage	ADHD, diagnosed at the age of 7 years Learning difficulties

Service providers included professionals from different sectors located at national, regional, and municipal care units. Table 2 provides an overview of the included service providers.

**Table 2:** Overview of service providers included in the study

Position	Location
Pediatrician (*N* = 1) Neuropsychologists (*N* = 6) Physiotherapists (*N* = 3) Occupational therapists (*N* = 3)	Child and Adolescent Habilitation unit, university hospital
Physiotherapists (*N* = 2) Occupational therapists (*N* = 2) Coordinators (*N* = 2)	Primary healthcare services, municipality 1
Physiotherapists (*N* = 2) Occupational therapists (*N* = 2) Coordinators (*N* = 2)	Primary healthcare services, municipality 2
Advisors from the school section (*N* = 2) Advisors from the kindergarten section (*N* = 2)	Pedagogical Psychological Service, municipality 1

In addition to the interviews, the families invited researcher SA to participate as an observer in planned coordination meetings at the respective children’s schools. Participants in these meetings were parents, service providers at children’s schools, and various other service providers central to the children’s and families’ long-term care. Two service providers from these coordination meetings had also participated in prior focus group interviews. Service providers were informed of and consented to researcher SA’s participation before the meetings.

### Data collection

**Fig. 1:**
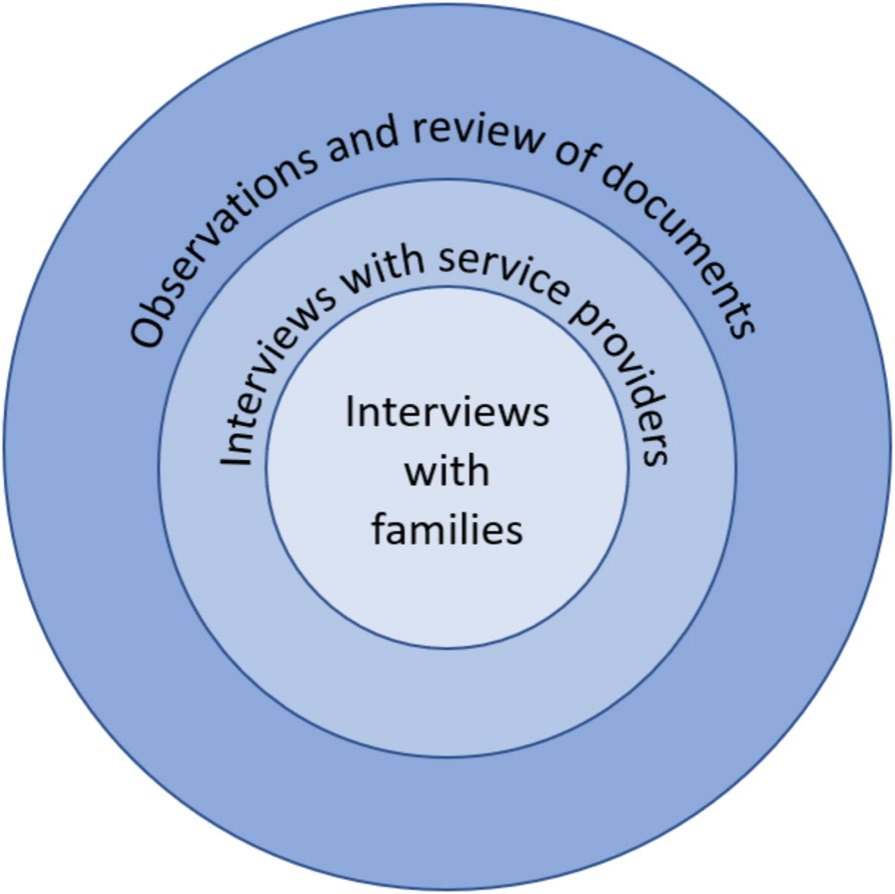
Data collection methods

Interviews with families were conducted prior to interviews with various service providers. Subsequently, the observations and a review of documents were carried out in parallel. The main data material was gathered through interviews with families and service providers, supplemented by data from observations and the document reviews, which provided additional, complementary information. Figure 1 provides an overview of the data collection methods.

### Interviews with families

Each family participated in three semi-structured interviews between April 2021 and December 2022. The interviews took place within four to eleven months and focused on services the children received because of their CP diagnosis and services that the families received to support their children. The first interview aimed to get to know each family, assess their experience of the services they received, identify stakeholders included in their long-term care, and determine families’ concerns, care needs, and preferences. Subsequent interviews focused on further elaborating any care and support activities that had occurred since the previous interview and addressing any other issues of concern or importance to the families. An open-ended approach was utilized, where questions to parents did not dictate whether they should discuss past or present experiences. This allowed them to freely address any topics they perceived as important throughout the child’s life. Children who could do so participated with their parents in parts of the interviews. Questions for the children were adapted to the individual child and the interview context [38–40]. The intervals between interviews, their location, length, and participants varied, considering families’ preferences, practical considerations, and Covid-19 restrictions. Interviews ranged in length from 60 to 120 min. Nine interviews were held in the families’ homes, seven were conducted digitally, and two were carried out at researcher SA’s office. In some cases, the child’s siblings were present at the interviews, whereas in a few cases, only one of the parents was present. At the end of the interview a summary of the discussion was given to allow participants to correct and supplement information. Researcher SA transcribed all family interviews.

### Focus groups with service providers

Focus groups were chosen for data collection with service providers to explore a wider range of views and perspectives [41, 42]. Six focus groups were conducted. Five included service providers who were colleagues in the same units, while one included service providers from two different municipalities who held the same positions. In addition to the focus groups, one individual interview was undertaken for practical reasons. Two focus groups had six participants, and four had four participants. A small group format was chosen to promote the active participation of all members and positively influence group communication [43]. Focus groups ranged in length between 108 and 148 min. The individual interview and one of the focus groups were conducted at researcher SA’s office, two focus groups took place at the service provider’s office, and three focus groups were conducted digitally. An interview guide with selected topics and introductory questions guided the focus groups to ensure the desired themes were discussed. At the end of the interview a summary of the discussion was given to allow participants to correct and supplement information. Two researchers were present in each focus group. Researcher SA transcribed all service provider interviews.

### Observations

Observations were conducted by researcher SA at four coordination meetings. During these observations, researcher SA introduced herself, explained the study’s purpose, and respond to any questions from the participants without taking an active role during the meeting [44, 45]. A semi-structured observation guide was utilized to gather information about the participants, their communication, discussed topics, perspectives addressed, children’s and families’ preferences, agreed collaboration, and the participant’s experiences of the meeting.

### Documents

The documents included in the review were identified through interviews with service providers, internet and literature searches, and assessments of the different services’ websites. These documents encompassed national regulations, guidelines and standards, regional procedures, along with information about the services. Additionally, they included national and Nordic reports detailing care needs and disparities in services for children with CP and their families. Prior to conducting the document search and review, we developed a search guide emphasizing regulations, delineation of responsibilities, anticipated collaboration, encountered challenges, and suggestions for service enhancement. Furthermore, we extracted details about the authors and target audiences of the documents, and identified any information not addressed [46].

### Analysis

Data were analyzed utilizing the stakeholder analysis approach, which involves 1) identifying stakeholders, 2) differentiating/categorizing stakeholders, and 3) investigating the relationships between stakeholders [47]. Triangulation of data collection methods comprising interviews, observations, and document review enabled the collection of in-depth information from several sources that informed all steps in the analysis, though different data sources formed the main component in each step [48]. During the analysis, information acquired through interviews and observations was discussed between the researchers to assess whether data saturation was obtained [49]. Information provided by the different families contained both similarities and differences. The same applied to the information given by service providers. Further methodological aspects are discussed in the strengths and limitations section of the discussion. Trustworthiness was achieved through triangulation of data collection methods, the involvement of several experienced researchers during the analysis, and by incorporating diverse perspectives through the inclusion of voices of multiple stakeholders [50]. To assist in the reporting of our qualitative research the COREQ checklist (COnsolidated criteria for REporting Qualitative research) was used [51].

#### Step 1: Identification of stakeholders

Stakeholders were identified through the analysis of interviews with families to determine their contact with service providers and the types of services offered to them. A list of stakeholders involved in each family’s long-term care was established, along with a table describing the services provided. This information was then collated and analyzed. Subsequently, transcribed interviews with service providers were analyzed to identify additional services, responsibilities, and relationships. To provide contextual information, documents were reviewed to identify organizations and service regulations. Stakeholders identified in the families’ long-term care were grouped into categories [52], as displayed in Table 3, along with information about their respective organizations.

**Table 3:** Identified stakeholders and their respective organizations and governing ministries

Stakeholders	Organization	Ministry and directorate
Child and Adolescent Habilitation 0–18 years *N* = 1	Specialist Healthcare services provided through the Hospital thrust in the Regional Health Authorities	Ministry of Health and Care Services and the Norwegian Directorate of Health
Local, national, and international hospital departments *N* = 18		
Tertiary care hospitals *N* = 6		
Private pediatrician *N* = 1		
General practitioner (GP) *N* = 1	Municipal Primary Healthcare services	
Municipal healthcare *N* = 7		
Private physiotherapist and chiropractor *N* = 1		
Assistive Technology Center *N* = 1	Governmental Labor and Welfare Administration	Ministry of Labor and Social Inclusion and the Norwegian Directorate of Labor and Welfare
Financial and social services provided through municipal Labor and Welfare offices *N* = 1		
Kindergarten and school *N* = 1	Municipal	Ministry of Education and Research and the Norwegian Directorate of Education
Pedagogical Psychological Service *N* = 1	County municipal	
Special Educational Service *N* = 1	Governmental	
Family Counselling Services *N* = 1	Municipal and governmental Child Welfare Services and Family Counselling Services	Ministry of Children and Families - The Directorate for Children, Youth, and Family Affairs - The Office for Children, Youth, and Family Affairs - County Governor
Child Welfare Services *N* = 1		

#### Step 2. Differentiate and categorize stakeholders

**Fig. 2:**
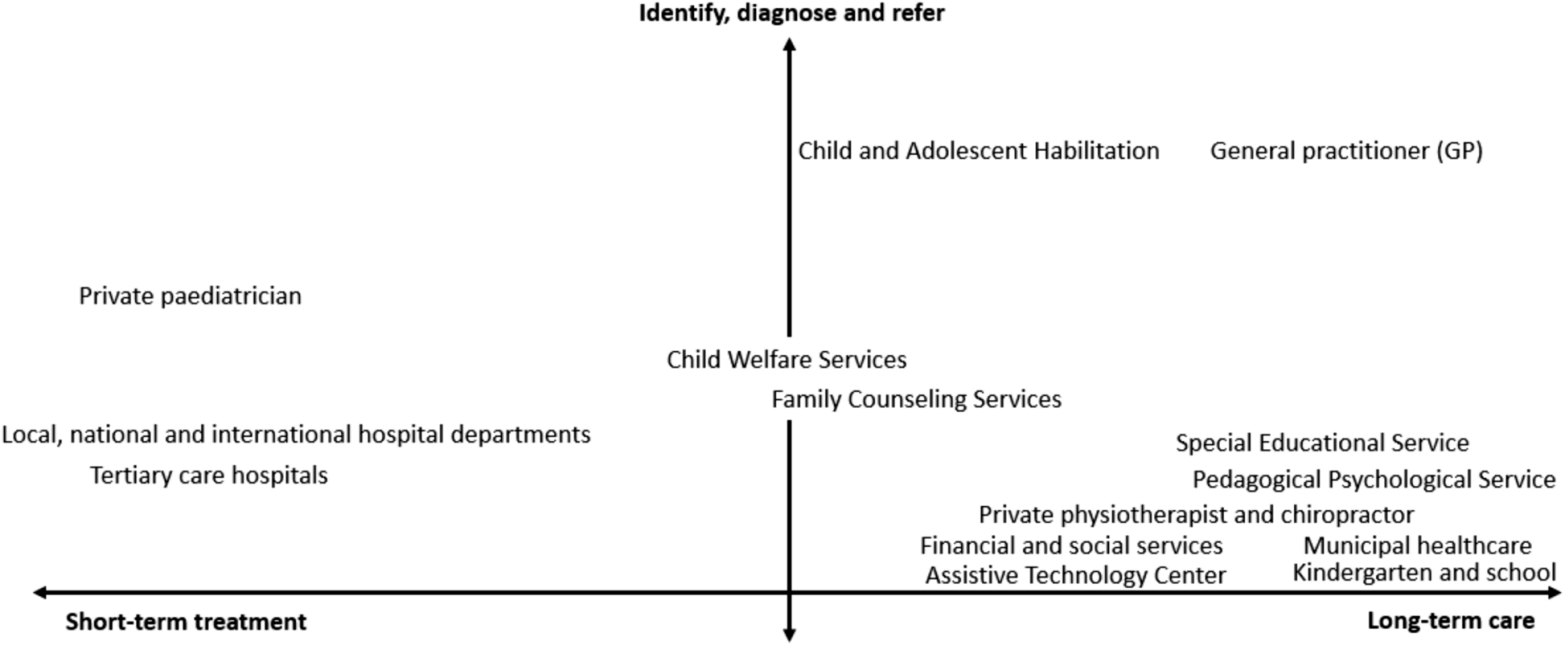
Stakeholder categorization according to their main responsibilities

During the second step, stakeholders were differentiated and categorized [47, 52] according to their main roles and responsibilities within services for children with CP and their families. Stakeholders who participated in interviews described their roles and responsibilities, while stakeholders who did not were categorized according to information provided by participating stakeholders, such as descriptions of services and collaborations. In addition, observations and document reviews aided the categorization of stakeholders. The stakeholder groups categorized according to their roles and responsibilities in offering services for children with CP and their families are depicted in a model in Fig. 2.

#### Step 3. Investigate relationships between stakeholders

**Fig. 3:**
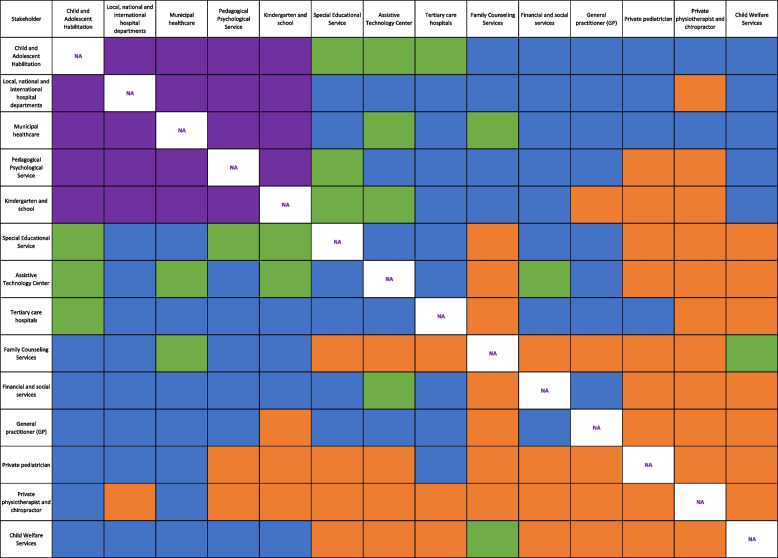
Actor-linkage matrix displaying the various relationships between stakeholders using color codes. Fully linked: purple; collaborative: green; communicative: blue; not linked: orange

To investigate the relationships between the identified groups of stakeholders [22], service provider interviews were analyzed. The different relationships were defined as *fully linked* (actors work together as a formal team with a mutual plan and shared resources to accomplish common goals), *collaborative* (actors work together as an informal team with specific responsibilities), *communicative* (actors share information only), and *not linked* (no contact between actors) [53, 54]. To visualize stakeholder relationships, an actor-linkage matrix was created, tabulating stakeholder groups in a two-dimensional matrix [47]. All stakeholders are listed in rows and columns, with the colored cells in the matrix representing different types of relationships between stakeholders [55]. The actor-linkage matrix is displayed in Fig. 3.

## Results

The results are presented according to the identified stakeholders involved in the care and follow-up of children with CP and their families, their roles and responsibilities, and the relationships between them.

### Identification of stakeholders

The interviews with families and service providers unveiled 42 stakeholders, consisting of 39 service units and three individual providers. These stakeholders were categorized into 14 groups: seven from the healthcare sector, three from the educational sector, two from the labor and welfare sector, and two from the sector for children and families. Table 3 presents an overview of stakeholders in each group, along with their organizational level with their affiliated governing ministry and associated directorate, involving various legislation and regulation of the services. Despite identifying 42 stakeholders in the interviews, the number involved in each child’s and family’s long-term care varied from 21 to 34. This variance is attributed to differences in the children’s CP gross motor function levels, associated comorbidities, and additional diagnoses, leading to distinct challenges, care requirements, and follow-up needs.

Throughout the interviews, families detailed the services they received for their child from birth to their current age, including changes in care needs, transitions in care, functional improvements, and emerging challenges over time. As such, the identified stakeholders entered the child’s and family’s long-term care at different periods and provided services over different time spans. For example, the Child and Adolescent Habilitation is the main care unit within the Specialist Healthcare services responsible for long-term follow-up during the period in which the child is 0–18 years old. For specific medical challenges, different local, national and international hospital departments including neurosurgery, neuropsychology, orthopedics, and Child and Adolescent Psychiatric units may provide treatment during shorter periods of time. This also includes tertiary care hospitals such as specialist rehabilitation units and the National Centre for Epilepsy. Further, long-term care included, amongst others, physiotherapy and various respite arrangements provided by both municipal healthcare services and private stakeholders, as well as agreed follow-up and facilitation in kindergartens and schools.

### Stakeholder categorization

The 14 stakeholder groups were differentiated and categorized according to their responsibilities for the services they provide to children with CP and their families. Although overlapping responsibilities were identified between some stakeholders the three central areas of responsibility were: 1) identifying, diagnosing, and referring, 2) providing short-term treatment, and 3) providing long-term care. Figure 2 summarizes the various stakeholders’ positions and places them in a three-dimensional model according to their main responsibilities for services offered to children with CP and their families.

#### Stakeholders responsible for identification, diagnosis, and referrals

This area of responsibility involves identifying and diagnosing newly occurring or escalating issues and making referrals to stakeholders in specialist and primary healthcare services, the educational sector, and the sector for children and families for further follow-up. Stakeholder groups within this category are the Child and Adolescent Habilitation unit, general practitioners (GPs), and private pediatricians.

The Child and Adolescent Habilitation unit comprises multidisciplinary specialists responsible for assessing and diagnosing CP and associated comorbidities. These may include somatic and cognitive conditions arising from the initial CP injury, complications linked to the CP diagnosis, or additional conditions that manifest as the child ages. According to established protocols in the national register and a follow-up program for children with CP, a CP follow-up appointment typically occurs annually or biennially. Additionally, separate intervals are set for cognitive assessment appointments to monitor conditions, detect changes, and enhance the child’s functioning. If challenges are identified during follow-up appointments, the Child and Adolescent Habilitation unit refers the child to other local, national, and international hospital departments, e.g. neurosurgery and orthopedics and tertiary care hospitals, e.g. specialist rehabilitation centers and The National Centre for Epilepsy for further treatment.

The GP’s responsibility includes identifying and assessing care needs during consultations with families and referring them to specialist and municipal healthcare providers. Specialist healthcare services may include referrals to Child and Adolescent Habilitation units and other local hospital departments, e.g., Child and Adolescent Psychiatry. Additionally, private pediatrician may refer the families to local and national, and international hospital departments as well as to tertiary care hospitals after considerations of families request during consultations. Referrals to stakeholders in specialist healthcare services due to challenges following the initial CP diagnosis are in the public services mainly the responsibility of the Child and Adolescent Habilitation units. They prefer to be the referring unit for treatment associated with the CP diagnosis and its related challenges to ensure that the treatment is appropriate, addresses all necessary concerns, and includes follow-up of the child and their family afterward.

#### Stakeholders responsible for providing short-term treatment

The two stakeholder groups, local, national and international hospital departments and tertiary care hospitals, classified within this category are mainly responsible for considering and treating specific areas within their expertise. These two groups include 24 stakeholders divided into three subgroups: a) stakeholders who evaluate and treat specific and defined areas, e.g., hearing and vision impairment, Botox injections, and adjustments of orthoses; b) stakeholders who evaluate and treat issues with a greater overall effect on the child’s function, e.g., orthopedic and neurological problems, such as hand, hip, and back issues requiring surgery, or muscles and tendons problems necessitating surgical lengthening, and c) stakeholders responsible for assessment and treatment to understand the child’s overall situation and potentially consider additional diagnoses, e.g., psychiatric treatment, rehabilitation, and diagnosis-specific assessments. Such treatment considerations often determine further healthcare needs, educational support, and social support.

#### Stakeholders responsible for long-term care

Stakeholders in this category are responsible for long-term care services according to the child’s and family’s needs. They can be divided into the following sub-groups according to services triggered by a) the family’s need for healthcare services and follow-up, b) the child’s CP diagnosis, associated comorbidity, and additional diagnoses, c) the child’s impaired functioning in kindergarten and school, and d) the need for family support. The family’s need for healthcare services and follow-up entails services from the GP, who, in addition to being responsible for identifying, diagnosing, and referring, is also in charge of follow-ups. As the family’s primary healthcare contact, they know the families’ overall use of services and help them coordinate and navigate within these services. Additionally, the GP coordinates medical care such as prescriptions and medication managements, often after medication trials have been initiated within specialist healthcare. Services offered to the children after CP diagnosis and further assessments within specialist healthcare include physiotherapy focused on e.g., continuing exercises and stretching of muscles and tendons, personal assistants and respite arrangements, chiropractic treatment, assistive technology, and financial and social services. Services triggered by the child’s impaired functioning in kindergarten and school include environmental facilitation and support of social interactions and assessments by the Pedagogical Psychological Service and the Special Educational Service. Based on these assessments, the municipality allocates additional pedagogical resources. Services triggered by the need for family support includes counseling provided by the Family Counselling Services and the Child Welfare Services in periods according to for example, challenging care situations.

### Relationships between stakeholders

The relationships between the 14 identified stakeholder groups involved in the children’s and families’ long-term care were analyzed using an actor-linkage matrix in Fig. 3. The relationships were classified as either fully linked (actors work together as a formal team with a mutual plan and shared resources to accomplish common goals), collaborative (actors work together as an informal team with specific responsibilities), communicative (actors share information only), or not linked (no contact between actors) according to degree and type of contact between stakeholders.

Most importantly, the children’s right to an assigned coordinator and individual care plan represents a fully linked relationship between stakeholder groups. An assigned coordinator from municipal healthcare contacts the included service providers to establish a responsibility group that aligns services provided to the child and family. Service providers in this group report the status of the child’s individual care plan through a digital tool and address status, challenges, and care needs in a coordination meeting twice a year. Through their shared responsibilities, the relationships between the child’s kindergarten or school, the Pedagogical Psychological Service, municipal healthcare, the Child and Adolescent Habilitation unit, and in some cases, the Child and Adolescent Psychiatric department at the hospital, are classified as fully linked.

Service providers offer a set of established measures to children and families that entail fully linked relationships between stakeholder groups. These measures are: 1) Clinic days at the Child and Adolescent Habilitation unit where the child and family meet internal physiotherapists, occupational therapists, pediatricians, and external pediatricians to discuss possible surgery and plan for follow-up activities. 2) Assistive devices days where children and families are provided information and test different types of supportive aids and equipment with help from physiotherapists, occupational therapists, and personnel from the Assistive Technology Center. 3) Consultation meetings between staff from the Child and Adolescent Habilitation unit and the hospital Child and Adolescent Psychiatric department to address children’s and families’ needs either during assessment for an additional diagnosis or before referrals.

Fully linked relationships between stakeholders also arise due to emerging children and family needs. When children and families experience challenges that have not been solved, an interdisciplinary team of participants from the Child and Adolescent Habilitation unit, the municipal coordinating unit, the Pedagogical Psychological Service, and the hospital’s Child and Adolescent Psychiatric department offer follow-up.

Collaborative relationships, where actors work as an informal team with specific responsibilities, also occur between stakeholders with fully linked relationships. This includes communication during referrals, assessments, distribution of responsibility, and alignment of follow-up activities. For example, a kindergarten or school refers the child to the Pedagogical Psychological Service when problems are identified, which collaborates with the kindergarten or school and the Child and Adolescent Habilitation unit to gather relevant information during their assessments. Subsequently, the kindergarten or school follows the recommendations provided by the Pedagogical Psychological Service. Other examples of collaborative relationships include the Child and Adolescent Habilitation unit, hospital departments, and municipal healthcare, which collaborate on preparation and examinations before surgery or facilitation of post-surgery training and exercise.

Additionally, the Special Educational Service collaborates with the Child and Adolescent Habilitation unit, the Pedagogical Psychological Service, kindergartens, and schools to provide information and training to facilitate alternative supplementary communication for the child. The Assistive Technology Center collaborates with kindergartens and schools on assistive devices and aids provided to the child. The municipal healthcare service collaborates with the Family Counseling Services, which also partners with Child Welfare Services to ensure the provision of family support.

Our analysis revealed several communicative relationships between stakeholder groups involving sending referrals to services or reports on assessments and treatment provided, in addition to recommendations for further follow-up. For example, the GP refers the child and family to the Child and Adolescent Habilitation unit and hospital departments for assessments and follow-up. Subsequently, the Child and Adolescent Habilitation unit and hospital departments send reports to the GP to communicate provided treatment, their assessments, and further recommendations. These relationships consist of information sharing without further conversations about responsibilities or the alignment of services. Additionally, some stakeholders are not linked while providing services to children and families.

## Discussion

This study identified stakeholders involved in integrated care for children with cerebral palsy and their families, including actors from the healthcare, educational, labor and welfare, and children and families’ sectors. These stakeholders are responsible for initial identification, diagnosis, referral, short-term treatment, and long-term care.

### Complexity of stakeholders

The children and families in this study interact with between 21 and 34 different stakeholders representing several different services, service levels and sectors. These stakeholders have a range of different roles and responsibilities and engage in a variety of relationships with both the families and each other in efforts to achieve integrated care for children with CP. The findings described here are indicative of a considerably complex care landscape that can be experienced as difficult to navigate by the families [56–58]. This is in line with previous research, which shows that families of children with chronic conditions often need follow-up from a large multidisciplinary network of service providers [59–61]. Among the providers in our study, a growing awareness of these complex care needs has resulted in newly implemented integrative measures developed because traditional measures were considered insufficient to meet children’s and families’ needs. These measures include cross-functional teams, consultation meetings to address responsibilities and follow-up possibilities, referrals, and assessment meetings. Additionally, these families commonly undertake extensive work to care for their children, with studies finding that their care burden is often greater than what is generally perceived by service providers [59, 62]. This means that families are in need of better support through easily accessible integrated services where the care burden is shared and coordinated between families and involved service providers [59]. In this context it is especially important to consider the role of various social determinants, such as for example social support, that might influence both the access, utilization and health and social outcomes, in the development of integrated services to children and families [63].

### Achieving integrated care

Achieving integrated care for children with CP and their families requires including the integrated care concept in policies, regulations, and procedures, embedded in management support, culture and values, and the individual service providers’ performance across sectors and levels [12, 64]. Our study on stakeholders’ organization (Table 3) and their responsibilities (Fig. 2) and relationship (Fig. 3) revealed that stakeholders from the healthcare sector and the educational sector work together in a fully linked manner through specific measures for integrated care. As such, important services for children and their families are integrated through the child’s responsibility group with regular coordination meetings, other established meeting arenas for stakeholders, and collaboration of follow-up services. Despite the implementation of measures for integrated care, research indicates that families of children with chronic illness and complex needs still experience unmet care needs [65, 66]. Additionally, service providers experience a lack of clarity about the integrated care model, including unclear areas of responsibility and poor communication between providers [67]. In particular, complex care needs that require simultaneous interventions involving service providers from different sectors and governmental levels are described as challenging to fully integrate [16].

Integrated care necessitates vertical integration between primary and secondary care, horizontal integration across health, educational support, and social care, and longitudinal integration of services spanning the lifespan [12, 13]. The results of this study showed vertical integration, where specialist and primary healthcare are connected; horizontal integration, where healthcare and educational support is connected; and longitudinal integration involving collaborative services for children aged 0–18 years. However, insufficient vertical integration was identified, where the GPs have only a communicative (information sharing) or no contact relationship with other stakeholders despite the GP`s explicit responsibility for follow-up. It could be argued that collaboration with other stakeholders is necessary for GPs to be aware of the families’ services, to assist with coordination and navigation within these services, and to coordinate medical matters. Insufficient horizontal integration was also identified, as one stakeholder group from the labor and welfare sector and all stakeholder groups from the children and families’ sector related to other stakeholders only through information sharing or had no contact at all. These stakeholders have important follow-up responsibilities in the children’s and families’ long-term care. Insufficient collaboration between sectors and levels is described in reports from a Nordic collaboration that aims to improve cross-sectorial services for children and families [14–16]. The Norwegian national guidelines for Collaboration on services for children, young people, and their families were implemented in 2022 to mitigate this issue [68].

Private provider stakeholders contacted by families are, by definition, not included in the public services, which may explain their lack of relationships with other (public) stakeholders. Similarly, for stakeholders responsible for treating specific and defined clinical issues only, their limited, clinically focused role may explain why their relationships with other stakeholders consist only of information sharing. However, stakeholders who provide services through common assessments and follow-up activities should reasonably be expected to collaborate more closely. This includes stakeholders responsible for identifying, diagnosing, and referring; stakeholders responsible for treating issues with a greater overall impact on children with CP; stakeholders responsible for assessment and treatment aimed to improve and support a child’s overall situation; and stakeholders responsible for follow-up, including municipal healthcare and educational services. Our results also showed that stakeholders who provide social care and family support offer important services from a long-term care perspective. They also need collaborative relationships with other stakeholders to become fully integrated service providers in integrated care for children with CP and their families.

Providing integrated services in the context of numerous stakeholders across sectors, regulations, institutional contexts, and levels (national, regional, municipal) requires cross-sectorial collaboration. Service providers from different sectors vary in educational background, experience, and knowledge, leading to different cultures, values, perspectives, and approaches [14]. Lack of knowledge regarding each other’s service areas and responsibilities may lead to a lack of communication between service providers. This can result in poor utilization of stakeholder colleagues’ competence and underutilized possibilities in follow-up services. Our results showed several situations with shared responsibility between stakeholders. For example, overall habilitation services are shared between the regional Child and Adolescent Habilitation unit, local, national and international hospital departments, tertiary care hospitals, and municipal healthcare services.

Similarly, coordination responsibility is shared between the regional Child and Adolescent Habilitation unit, local, national and international hospital departments, the GP, and municipal healthcare. This distribution of responsibility is also described in the Norwegian Regulations on habilitation, rehabilitation, and coordination, which aims to strengthen the interaction between service providers, patients, and carers and between service providers across sectors and levels [34]. Shared responsibility for service provision between stakeholders with different responsibilities, competence, perceptions of problems, and angles on solutions can contribute to multidimensional and holistic approaches. However, without guidelines for approaching and organizing follow-up activities, shared responsibilities can also lead to stakeholders disclaiming liability for services not being provided as intended. Further empirical research is needed that explores providers as well as families’ experiences of collaboration among the stakeholders involved in health and social care services for children with CP and how such services should be organized to ensure integrated care.

### Strengths and limitations

A strength of this study was the triangulation of methods comprising interviews, observations, and document review that enabled the collection of in-depth information from several sources. Additionally, the families’ participation in longitudinal interviews enabled information about the progression of their long-term care and allowed for the follow-up of interview topics over time. Service providers’ affiliation with various sectors, levels, and units offered information from diverse perspectives, providing a broad exploration of the services affecting children with CP and their families.

A limitation of the study was that only families with children up to the age of 12 years were included and, as such, they were not in a position to discuss the care over time for older children, including adolescents, and young adult. The inclusion of more families and children representing a wider age range could have led to different findings, for example the identification of additional stakeholders. Future research should explore broader experiences of long-term care conditions, also among families with children of different age groups including older children, adolescents, and young adults. Another limitation of this study was that not all service providers identified by the families in the initial round of data collection were interviewed in subsequent rounds due to time restrictions and a lack of access to and availability of those stakeholders. Therefore, stakeholders such as general practitioners and a wide range of medical specialists have not been included in this study. Future research should additionally consider the voice and perspective of those stakeholders.

## Conclusion

Long-term care for children with CP and their families is complex, involving numerous stakeholders across various care units, levels, and sectors, governed by regulations within different institutional contexts. Responsibilities may overlap and lack clarity, and their relationships are fully integrated only to a certain degree. Stakeholders’ affiliation with different sectors and their varying roles, responsibilities, and relationships can strengthen and contribute to a holistic approach. However, without clear guidance facilitating cross-sectoral collaboration between services at both individual and system levels, alongside close collaboration with families, given situations and approaches may lead to a lack of collective understanding and unmet needs for children and families. This indicates the need for further research on collaborative experiences among children, families, and service providers, and their impact on integrated services.

## Supplementary Information

Supplementary Material 1.

Supplementary Material 2.

Supplementary Material 3.

Supplementary Material 4.

Supplementary Material 5.

Supplementary Material 6.

## Data Availability

The datasets generated and analyzed during the study are not publicly available due to confidentiality but are available from the corresponding author upon reasonable request and pending ethics clearance from the Regional Committee for Medical and Health Research Ethics in Norway and the Norwegian Agency for Shared Services in Education and Research.

## Abbreviations

CP: Cerebral Palsy
GMFCS level: Gross Motor Function Classification System level
ADHD: Attention Deficit/Hyperactivity Disorder
GP: General Practitioner
