# Supplementary material for: Exploring integrated care for children with cerebral palsy: a stakeholder analysis
Source: BMC Health Serv Res. 2025 Jul 7;25:936. doi: 10.1186/s12913-025-13015-x (PMC12232699; doi:10.1186/s12913-025-13015-x)
Supplement: Supplementary file 1 — Supplementary Material 1. [file 12913_2025_13015_MOESM1_ESM.docx]

# Interview guide for parents

## First interview

The first interview aims to get to know the family and create a good relationship that lays the foundation for further collaboration. The following interviews seeks information about the family’s concerns, care needs, preferences, services included in long-term care for their child, and their experiences and perspectives on these services.

### Introductory question

- How do you experience everyday life with a child with cerebral palsy?

### Follow-up questions

- What health and care services do you receive for your child, and what service providers do you have contact with?
- Which services and providers are the most important for you in the long-term care for your child?
- Can you tell me about your experiences with the services?
- Do you feel that the services are adapted to the family’s needs and if so, how?
- What is important for you to experience the services as adapted to the family's needs?
- How do you experience your collaboration as parents with service providers?

## Follow-up interviews

The follow-up interviews seek to further explore themes identified in the first interview, get to know the child and the family's history better, and follow events in their long-term care. The questions outlined below form the basis for all follow-up interviews. Questions in the specific interviews will vary and will be adapted according to the events in the families' long-term care and areas of importance to them at the time of the interview.

## Introductory questions

- Can you tell me what has happened since I last spoke with you?

## Follow-up questions

- Can you tell me about your experiences from meetings and collaboration with service providers?
- What role do you, as parents, want to play in collaboration with service providers regarding involvement, decisions, and responsibility?
- What role do you want your child to play?
- Can you tell me how service providers include you and your child in the services?
- How can service providers ease your follow-up role/responsibility regarding the services for your child (treatment, appointments, periods with assessments and rehabilitation, follow-up at school, weekly/daily exercise)?
- In what way do you feel that the services offered have a long-term perspective where future challenges and needs are addressed and planned for?
