# Supplementary material for: Exploring integrated care for children with cerebral palsy: a stakeholder analysis
Source: BMC Health Serv Res. 2025 Jul 7;25:936. doi: 10.1186/s12913-025-13015-x (PMC12232699; doi:10.1186/s12913-025-13015-x)
Supplement: Supplementary file 2 — Supplementary Material 2. [file 12913_2025_13015_MOESM2_ESM.docx]

# Interview guide for children

The interviews have a conversational format where the child is allowed to tell his/her story. The aim is to clarify the child's encounters with the services and the child's experiences and perspectives. Questions will be adapted according to age level, maturity, and how the conversation evolves. The conversation will be held on the child's terms and address topics the child is comfortable discussing.

## First interview

The conversation will start with an introductory question asking the child to talk about the last day at school. The questions’ purpose is to start the conversation with something the child can easily talk about and to create a safe framework for the conversation. Additionally, the question can provide information about services/follow-up the child receives during everyday life at school.

### Introductory questions

- Can you tell me about the last day/yesterday at school? Can you tell what happened right from the time you came to school?

I will then ask the child to tell me about his/her last meeting with the services/service providers. The purpose of the question is to obtain information about how the child experienced both the meeting and the services. The question is formulated based on information from the parents about the services the child receives and the service personnel with whom the child interacts.

### Follow-up questions

- Can you tell me about the last time you met someone (service provider) who wanted to talk to you about your health and well-being or carry out examinations, tests, or exercise together with you?

## Follow-up interview

The follow-up conversation seeks to get to know the child's history and further explore topics the child discussed at the first meeting. The conversation starts with an introductory question about what has happened since we last spoke. Follow-up questions will be adapted based on the information obtained during the first conversation with parents and the child (e.g., follow-up by a physiotherapist, training groups or rehabilitation periods, meetings concerning assessments or treatment).

### Introductory questions

- Can you tell me about what has happened since we last spoke?

### Follow-up questions

- Can you tell me what happened at this meeting/appointment (the question will be adapted based on the information the child provided in the previous interview)?
