# Supplementary material for: Exploring integrated care for children with cerebral palsy: a stakeholder analysis
Source: BMC Health Serv Res. 2025 Jul 7;25:936. doi: 10.1186/s12913-025-13015-x (PMC12232699; doi:10.1186/s12913-025-13015-x)
Supplement: Supplementary file 3 — Supplementary Material 3. [file 12913_2025_13015_MOESM3_ESM.docx]

# Interview guide for focus groups with service providers

The interview questions will address the following:

- Service providers' roles, responsibilities, and tasks
- Collaboration with families
- Collaboration between the services/service providers

## Introductory question

- What do you do when you receive a referral of a child and family who need services from you?

## Follow-up questions

- What do you do to meet the family's care needs?
  - Methods and adaptations
- What is your experience of including the family in the services (collaboration)?
  - Can you tell us about situations when the collaboration was successful versus challenging?
- What do you think is important to achieve an effective collaboration with the family?
  - Barriers and facilitators?
  - Improvement areas and opportunities?
- How would you describe the collaboration between the service providers involved in the family’s long-term care?
  - Which internal and external service providers do you collaborate with?
  - How often do you have contact?
  - How would you describe the contact?
  - Can you tell us about situations when the collaboration and was successful versus challenging?
- What do you think is important to achieve effective collaboration between service providers who follow up with the family?
  - Barriers and facilitators?
  - Improvement areas and opportunities?
