# Supplementary material for: Exploring integrated care for children with cerebral palsy: a stakeholder analysis
Source: BMC Health Serv Res. 2025 Jul 7;25:936. doi: 10.1186/s12913-025-13015-x (PMC12232699; doi:10.1186/s12913-025-13015-x)
Supplement: Supplementary file 4 — Supplementary Material 4. [file 12913_2025_13015_MOESM4_ESM.docx]

# Guide for observations

## Observations in multidisciplinary coordination meetings

Observation will be carried out in multidisciplinary coordination meetings at the children’s school. The researcher will present herself, the study, and the aim of the observation and respond to questions from the participants.

## The observations aim to gather information about the following:

- Which services and service providers participate in the meetings?
- Is there a planned agenda for the meeting?
- Are participants prepared for the meeting?
- Who is sharing the meeting?
- Who writes the minutes of the meeting?
- Which topics are discussed?
- How are the families’, parents’, and children’s concerns, experiences, and care needs addressed?
- In which order are the topics addressed?
- How does the participants collaborate to establish follow-up activities that meets the families’ care needs?
- How are the perspectives of the various participants addressed?
- How are possible disagreements addressed and aligned?
- How is information shared between participants before, during, and after the meeting?
- Does the meeting conclude with specific and agreed plans for further follow-up activities?
- How are planned follow-up activities documented and shared between the participants?
- Is the next coordination meeting scheduled?
- How do the participants experience the meeting?
