# Supplementary material for: Exploring integrated care for children with cerebral palsy: a stakeholder analysis
Source: BMC Health Serv Res. 2025 Jul 7;25:936. doi: 10.1186/s12913-025-13015-x (PMC12232699; doi:10.1186/s12913-025-13015-x)
Supplement: Supplementary file 5 — Supplementary Material 5. [file 12913_2025_13015_MOESM5_ESM.docx]

# Search guide to review documents

Documents in the review will be identified through interviews with service providers, internet searches, literature searches, and reviews of the different services’ websites. These documents include national regulations, national guidelines and standards, regional procedures, information about the services, and national and Nordic reports describing care needs and discrepancies in services for children with CP and their families.

The document review aims to gather information about

- How do the ministry and directorate regulate the services?
- Which laws, regulations, and guidelines apply to the services?
  - How is the various service providers' responsibility outlined?
  - How is the expected collaboration between the services described?
- What do the service providers' procedures describe?
  - Responsibility and expected collaboration?
- How are the care needs of children with CP and their families described in the documents?
- Which discrepancies in the services are described?
- How is the expected collaboration between services and service providers described?
- What are recommendations for improvement within the services?
- Who is the author of the documents?
- Who is the intended audience of the document?
- What does the document not describe?
